# Gender beliefs and norms underlying intimate partner violence stigma among women living in Botswana: Results of an exploratory factor analysis

**DOI:** 10.1371/journal.pgph.0004113

**Published:** 2025-02-05

**Authors:** Ari Ho-Foster, Mercilene Tanyaradzwa Machisa, Lorato Ruth Moalusi, Nicola Christofides

**Affiliations:** 1 Faculty of Medicine, University of Botswana, Gaborone, Botswana; 2 School of Public Health, Faculty of Health Sciences, University of the Witwatersrand, Johannesburg, Gauteng, South Africa; 3 Gender and Health Research Unit, South African Medical Research Council, Johannesburg, Gauteng, South Africa; 4 Botswana Gender Based Violence Prevention and Support Centre, Gaborone, Botswana; St John's Medical College, INDIA

## Abstract

Gender inequitable beliefs, blaming attitudes, externalised and internalised stigma are commonly recognised barriers for intimate partner violence (IPV) survivors seeking help. However, the measurement of IPV stigma, its associations with inequitable gender beliefs and impacts on survivor disclosure, help-seeking behaviours, and mental health outcomes remain understudied. We explored women’s agreement with statements about gendered power dynamics and violence in intimate heterosexual relationships, before identifying and psychometrically testing scales derived for measuring community norms and beliefs underlying stigma to IPV. We used data from a nationally representative sample of 596 women living in Botswana. Exploratory factor analysis (EFA) occurred with responses of IPV survivors, and involved items from the Community Ideas about Gender Relations, Community Ideas about Rape, and Gender Equitable Women Scales. For each EFA identified scale, we estimated reliability (McDonald’s omega (**ω**)) and correlation with psychosocial outcomes related to IPV stigma. Among IPV survivors, we also considered whether survivors had disclosed their experience of abuse to others prior to the interview. Some 40.9% (n = 244) of women have experienced physical and/or sexual IPV at least once in their lives. Among them, an EFA of 31 gender beliefs and norms identified three latent variables: community norms about male dominance over female partners (C-MDP) (11 items; **ω** = 0.86); respondent beliefs about male dominance over female partners (I-MDP) (12 items; **ω** = 0.83); and survivor blaming attitudes (SBA) for the IPV they experienced (8-items; **ω** = 0.83). Some 15% of survivors had attempted suicide in the past, 8% had disclosed having suicidal thoughts, 49% were considered at risk for depression, and 18% at risk for post-traumatic stress disorder. Survivors who more strongly endorsed C-MDP appeared more likely to have attempted suicide (p = 0.04), and less likely to have disclosed their IPV experience prior to the study (p = 0.002). Survivors who more strongly endorsed SBA appeared more likely to have had suicidal thoughts (p = 0.02) and greater post-traumatic stress symptoms (p = 0.06). C-MDP, I-MDP and SBA appear related to psychosocial and disclosure outcomes. Gendered social norms may play an important role in understanding how survivors experience IPV stigma. We recommend further research into culture-informed practices that act to socialise such norms.

## Introduction

Stigma can make it challenging for survivors of IPV to seek help. Goffman [1] explained stigma as a way society looks down on people because of certain traits that are viewed as shameful or discrediting [2]. Link and Phelan further explained that stigma involves labelling, stereotyping, separation, leading to loss of respect and discrimination, particularly when there are power imbalances [3].

Overstreet and Quinn proposed a model to explain how stigma affects IPV survivors and prevents them from seeking help. They initially described three forms of stigma: (i) Cultural stigma refers to societal beliefs and stereotyping that make IPV survivors feel their experiences are less valid or important. (ii) Internalised stigma refers to when survivors start to believe the negative stereotypes about them are true. (iii) Anticipated stigma includes fears of being treated negatively by others if they learn of the survivor’s abuse [4]. Two additional forms of stigma were later added: (iv) Enacted stigma describes discrimination and prejudice that survivors experience from others. (v) Perpetrator stigma refers to emotionally, verbally, and/or psychologically harmful messages that survivors receive directly from their abuser [2]. Their model further describes stigma in terms of: blame, discrimination, loss of status, isolation, and shame. Several IPV researchers [5,6] have further distinguished self-blame into two forms: behavioural and characterological self-blame [7]: Behavioural self-blame for the abuse is attributable to modifiable aspects of a survivor’s behaviour, while characterological self-blame relates to aspects of the survivor that they cannot change and less within their control [7].

Survivors of IPV in Botswana likely face stigma as other women in the region. In neighbouring South Africa, Willan et al. studied feelings of shame, self-blame, and internalised stigma among female rape survivors. They discovered that these survivors felt stigma personally, but this was often influenced by their interactions with family, community members, and service providers [8]. In their study of rape stigma, Jewkes et al. described stigma in two forms: external stigma – feelings of ‘social devaluation’, or discrediting of rape survivors – and internal – shown through expressions of self-blame and/or shame for being raped that ultimately reflect self-devaluation [9]. While assessing these forms using a local HIV stigma scale adapted for rape stigma [10], they found about half of their respondents had experienced stigma from others (external), and more than one-third agreed with statements that reflected internalised stigma [9]. As rape stigma excludes physical, psychology and other aggressive behaviours that can comprise IPV, its estimation likely underestimates the prevalence of general IPV stigma.

As well, local social, cultural and gender norms influence how women IPV survivors experience stigma in their homes and communities. Yang’s theory of “What Matters Most” (WMM) offers a framework to understand stigma within a cultural group [11–13]. In studies conducted in Botswana and elsewhere, Yang et al. examined stigma within cultural groups and found group members engaged in daily life to fulfil WMM to them towards achieving ‘full status’ [11,14–17]. Among participants in Botswana, Yang et al. identified having children, competence in caring for children, and being the foundation of a household that takes care of the ‘home, husband, and kids’, as ‘cultural capabilities that ‘matter most’ to womanhood [16]. They also noted that stigma is experienced most strongly “when people are not able to participate in the activities that ‘matter most’ and determine ‘personhood’ in their culture” [18]. So, understanding the complex cultural factors affecting gender dynamics is critical to grasp the experiences of women dealing with IPV stigma in their daily lives.

In Botswana, gender norms remain a major barrier to women’s access to IPV services. A gender analysis of Botswana by Ramatlala, Bloom and Machao found, through cultural norms pervasive in parts of society, women accepted conditions of marriage that included: obedience to her husband and being beaten by a man as normal and acceptable [19]. Dube et al. explain that the traditional practice of paying ‘bride wealth,’ known as *bogadi* or *lobola*, is seen as a way to validate a traditional marriage. They argue that it symbolizes gratitude to the wife’s parents for raising their daughter, rather than being a method of buying a wife, which some people claim [20]. However, some groups in Botswana have viewed *bogadi/lobola* as exchanged when a woman accepts male dominance and surrenders ‘reproductive power’ to her new husband [21]. This brings to question a woman’s right to refuse sex in a marriage. Married IPV survivors who seek help are further challenged as they face a legal framework that only recently recognised the rights of women within marriage and does not include marital rape within the current definition of sexual assault [22,23].

With these definitions and contexts in mind, we considered women’s agreement with a series of statements about norms and beliefs. These statements, used by Southern scholars to measure victim-blaming attitudes, power and violence in heterosexual relationships, were captured in the Botswana Gender Based Violence Indicators Survey (2012) [24]; these statements may also reflect norms and beliefs that shape how stigma is experienced by female IPV survivors. We explored their psychometric properties and identified potential underlying factors. Where possible, we then developed scales for each underlying factor and tested each scale for convergence with key mental health outcomes.

## Methods

Setting: Botswana is a southern African nation that is home to 2.37 million residents. Among women in Botswana, studies have estimated that 49%–62% of adult women have experienced some form of IPV in their life; 19%–29% disclosed experiencing it in the past 12-month [24–26]. The link between experience of IPV and poorer health outcomes among women is well established [27], including greater vulnerability to HIV infection [28–30]. Studies have confirmed a gap between IPV experience and getting help also exists in Botswana. A 2012 report by Machisa and van Dorp comparing administrative data from police records and population-based surveys also found estimates derived from police report to underestimate greatly the prevalence of IPV (0.66% by police report vs. 28.9% by community-based survey) [24].

We analysed data captured through the first nationally representative stratified random sample of women participants of the BGBVIS in 2011 [24]. It collected data in an era prior to wide recognition of IPV as pervasive in Botswana, and prior to the advent of social movements (e.g. #MeToo) supporting survivor disclosure in the country. As such, the data arguably was collected at a time when survivors faced greater stigma disclosing experiences of abuse, allowing the investigators a unique opportunity to understand the influence of gendered subjective norms.

Sampling: A nationally representative sample of adult women in Botswana was obtained using a multi-stage random sampling design. Primary Sampling Units (PSUs) were randomly selected from the 2011 census, followed by random selection of households within PSUs. One eligible adult female per household was invited to participate. A total of 639 women consented to participate in the study between 11/11/2011 and 23/11/2011, corresponding to an overall response rate of 85% [24]. Further details of the approach to sampling and data collection are published elsewhere [24]. Data was accessed for research purposes on 30/05/2023. For this secondary analysis, we included 596 women who had ever been in a relationship and who had responded to questions concerning experience of IPV per WHO VAW measures [31].

### Ethics

Permission to carry out this study was obtained from the Institutional Review Boards of the University of Botswana (UBR/RES/IRB/BIO/342), the University of the Witwatersrand (M230227 MED23-01-053), and the Government of Botswana (MYSC 9/1/1). Primary data collection was approved by the Botswana Ministry of Health Research and Development Division (PPMEE 13/18/1 PS IV (37)); permission to access community research sites was obtained through a process of community mobilisation described elsewhere [24]. The primary study team collected data in accordance with the WHO Ethical and Safety Recommendations for Research on Domestic Violence against Women [32]. Participants provided written informed consent to participate in the study. Data for the primary survey were collected by trained female researchers who adhered to established safety protocols for violence research, including privacy, confidentiality, and participant safety. Participants self-completed a structured questionnaire in English or Setswana using Personal Digital Assistants (PDAs), with the option to skip questions at their discretion [24].

### Inclusivity in global research

Additional information regarding the ethical, cultural, and scientific considerations specific to inclusivity in global research is included in the Supporting information (S1 Checklist).

### Measurement

Socio-demographic characteristics measured in this study: age and education level of the participant; if the participant indicated working in the past 12 months, and, if so, the type of work; household food insecurity; and household size.

IPV experience and disclosure: We defined *IPV survivors* as those women who had disclosed any lifetime experience of physical and/or sexual violence by a current or former intimate partner during the study. Among survivors, we characterised a participant as having *disclosed abuse to someone* PRIOR to the study, if: they had indicated disclosing experiencing abuse to the police, to a family member, or while receiving medical attention; if they went to a shelter, or if they had spoken out publicly about the abuse.

Gender attitudes and beliefs scales: We explored endorsements of 33 selected statements about power dynamics and violence within intimate heterosexual relationships from three scales:

(i) From the Community Ideas about Gender Relations scale measures, we considered ratings of 22 items that elicit normative and personal perceptions of power dynamics in heterosexual marriage, including: “a woman should obey her husband”; “if a woman works she should give her money to her husband”; “a man should have the final say in all family matters”; “a woman needs her husband’s permission to do paid work”; “a woman cannot refuse to have sex with her husband”; “children belong to a man and his family “; “there is nothing a woman can do if her husband wants to have girlfriends”; “if a wife does something wrong her husband has the right to punish her”; “if a man has paid *lobola* for his wife, he owns her”; “if a man has paid *lobola* for his wife, she must have sex when he wants it”; and, “if a man beats you it shows that he loves you” [28,33,34].
(ii) From the Community Ideas about Rape scale measures, we considered ratings of 8 items eliciting normative and personal views that: “when a woman is raped, she is usually to blame for putting herself in that situation”; “in some rape cases women actually want it to happen”; “if a woman doesn’t physically fight back, it’s not rape”; and, “in any rape case one would have to question whether the victim is promiscuous” [28,33,34].
(iii) Finally, from the Gender Equitable Women Scale, we included ratings of agreement with three statement concerning power dynamics between intimate partners: “There are times when a woman deserves to be beaten”; “it is a woman’s responsibility to avoid getting pregnant”; and, “my husband or boyfriend would be outraged if I asked him to use a condom” [35,36]. We excluded from analyses items of the scale not specifically related to a patriarchal power difference in the relationship between intimate partners: e.g., sharing of housework, male stereotypes around strength and sexual readiness, and childcare responsibilities.

Mental health variables measured included: disclosed having ever attempted suicide (“Have you ever attempted suicide because of violence you’ve experienced in your life?”); suicidal ideation in the past month (“…about the past four weeks, has the thought of ending your life been in your mind?”); greater depressive symptoms (Center for Epidemiologic Studies Depression Scale (CES-D)) [37]; and greater symptoms of post-traumatic stress (modified form of the Harvard-Trauma Questionnaire summary score) [38]. To assess clinical significance, we defined participants: as at risk for clinical depression based on a CES-D score of 16 or higher [39], and at risk for Post-traumatic stress disorder (PTSD) based on a Harvard-Trauma Questionnaire average score of 2.5 or higher [38–40].

### Psychometric analyses

We anticipated IPV survivors experienced stigma to IPV differently from non-survivors and that their perceptions of gender-conditioned violence was different from those who had not experienced IPV. To account for this heterogeneity, we first developed an initial measurement model using exploratory factor analysis exclusively among IPV survivor responses. We calculated Bartlett’s test of sphericity (p-value < 0.05) to assess the suitability of the set of variables for data reduction [41], and Kaiser-Meyer-Olkin (KMO) (>0.5) to confirm the included dataset variables were adequate to perform exploratory factor analysis [42]. We performed a principal component factor analysis to explore underlying latent variables linked with the items. We considered Velicer’s minimum average partial correlation approach to determine the number of factors to extract [43,44]. Towards achieving a simplified structure, we considered several oblique rotations to allow for any correlation observed between factors [45]. We report results following a Promax oblique rotation, as its loading transformations appeared the most simplified. We observed factor loadings of 0.3 or more as indicative that an item was loaded on a particular factor, and excluded any items that did not load on any of extracted factors at that threshold. We assigned any items cross-loaded (loading >0.3 on multiple extracted factors) after considering semantic consistency of the cross-loaded item with other items in the factor, and strength of the loading. Among factors identified, internal consistency (reliability) was assessed using McDonald’s Omega [46,47].

With the items loaded on factors extracted among the IPV survivors, we calculated the loadings and internal consistency for the same items among non-survivors.

For each identified factor, we then calculated scale scores by summing the total response value for each statement (1 = strongly disagree, 2 = disagree, 3 = agree, 4 = strongly agree), assigning higher response values as greater endorsement of the underlying factor. For example, strong agreement with the statement “My community thinks that if a man has paid *lobola* for his wife, she must have sex when he wants it” suggests the respondent perceives the norms of her community favour male power over their partners. Among IPV survivors, she may also anticipate greater stigma from her community if she were married and reported experiencing forced sex from her husband.

Using the constructed scale scores, we then assessed construct validity, testing a number of hypotheses (H). Concerning known group validity, we anticipated stigma to IPV would be felt more strongly among IPV survivors than non-survivors. As such we hypothesized that endorsement of scales measuring factors of IPV stigma experience would be stronger among IPV survivors compared with non-survivors (H1).

Concerning discriminative and convergent validity, we derived hypotheses based on prior studies [9,48]. Among IPV survivors among whom IPV stigma would be considered more salient, we proposed higher scale scores would appear positively associated with negative psychosocial outcomes, including: greater risk of attempting suicide and suicidal ideation; greater depressive symptoms; and greater symptoms of post-traumatic stress (H2-H5). Among women who had not disclosed prior experience of physical or sexual partner violence (non-survivors), we proposed that IPV stigma would be less salient, and so hypothesized associations between IPV stigma scales and adverse psychosocial outcomes would be less strongly associated among non-survivors compared with survivors (H6-H9).

Finally, among survivors, we hypothesized that higher scale scores would appear negatively associated with having disclosed abuse to someone prior to the study (H10).

We assessed for normality of continuous variables using Shapiro – Wilk W test [49,50]. Differences in distribution of continuous variables were assessed using unpaired t-test if normally distributed, and Wilcoxon Rank-Sum test if not normally distributed. We estimated Spearman’s rank correlation coefficient (ρ) to assess for correlations between each scale identified and ordinal or continuous outcomes [51].

To assess the influence of missing data, we performed a sensitivity analysis comparing results of the naïve analysis with one involving a dataset completed by single imputation. For each candidate scale item variable, we imputed values generated from the mean (rounded up to the next whole number) of the known values of the variable. As we hypothesised the interpretation of a majority of the scale items would differ based on prior experience of physical and sexual violence, we estimated mean separately for survivors and non-survivors before replacement under missing completely at random assumptions. Using scale scores derived from imputed values, we then repeated analyses to assess for convergent validity with psychosocial outcomes. See S1 File: Sensitivity analysis assessing the influence of missing data, including Stata code.

We assessed statistical significance at the 5% level and performed analyses using Stata 17.0 [52].

## Results

This study reports analyses of a subset of the 639 women who participated in the BGBVIS, for whom data was available to assess lifetime exposure to physical or sexual IPV for 596 women who had been in prior relationships.

Nearly all (98.7%, 588/595) identified as Black African and citizens of Botswana (94.6%, 564/596), and most indicated they were Christian (89.6, 533/595). Two of every five women (40.9%, 244/596) indicated experiencing physical and/or sexual IPV at some point in their lives. As expected, symptoms of depression (p < 0.001), having attempted suicide (p = 0.003), and post-traumatic stress (p < 0.001) each appeared more common among IPV survivors, compared with non-survivors (see Table 1).

**Table 1:** Demographics.

Characteristic	Overall (n (%))	Experienced physical and/or sexual violence by an intimate partner ever in their lives?		p-value
		Yes (n (%))	No (n (%))	
Overall	596 (100)	244 (40.9)	352 (59.1)	–
Age in years				0.11
18–29	249 (41.8)	111 (45.5)	138 (39.2)	
30–44	194 (32.5)	81 (33.2)	113 (32.1)	
45+	153 (25.7)	52 (21.3)	101 (28.7)	
Education: Level of schooling completed				0.52
Less than high school	358 (60.2)	143 (58.6)	215 (61.3)	
At least high school	237 (39.8)	101 (41.4)	136 (38.7)	
Missing (n)	1	0	1	
Worked in the last 12 months?				0.32
No	349 (58.6)	137 (56.2)	212 (60.2)	
Yes	247 (41.4)	107 (43.8)	140 (39.8)	
If yes, type of work?				0.60
Police, security, or armed forces	19 (7.7)	9 (8.5)	10 (7.2)	
Professional	48 (19.6)	20 (18.9)	28 (20.1)	
Domestic worker	57 (23.3)	21 (19.8)	36 (25.9)	
Driver/transportation	1 (0.4)	1 (0.9)	0 (0.0)	
Other	120 (49.0)	55 (51.9)	65 (46.8)	
Missing	2	1	1	
Reside in a home where people sometimes/often go without food?				0.20
Often/Sometimes	283 (47.6)	123 (50.8)	160 (45.5)	
Seldom/Never	311 (52.4)	119 (49.2)	192 (54.5)	
Missing	2	2	0	
Household size				0.55
1	29 (4.9)	11 (4.5)	18 (5.1)	
2–5	312 (52.4)	121 (49.8)	191 (54.3)	
6–10	198 (33.3)	89 (36.6)	109 (31.0)	
10+	56 (9.4)	22 (9.1)	34 (9.7)	
Missing	1	1	0	
Attempted suicide in the past				0.003
No	509 (89.1)	202 (84.5)	307 (92.5)	
Yes	62 (10.9)	37 (15.5)	25 (7.5)	
Missing	25	5	20	
Thought about ending life in past month				0.21
No	538 (93.2)	221 (91.7)	317 (94.4)	
Yes	39 (6.8)	20 (8.3)	19 (5.7)	
Missing	19	3	16	
Depressive symptoms (CES-D) – median score (IQR)	12 (5–22)	15 (8–27)	9 (4–17)	<0.001
At risk for clinical depression (CES-D≥16)				<0.001
No	383 (64.3)	125 (51.2)	258 (73.3)	
Yes	213 (35.7)	119 (48.8)	94 (26.7)	
Post-traumatic stress – median score (IQR) (HTQ Section 3)	1.4 (1.1–1.9)	1.57 (1.2–2.03)	1.27 (1.03–1.68)	<0.001
At risk for PTSD (Avg HTQ raw score ≥2.5)				0.44
No	497 (83.4)	200 (82.0)	297 (84.4)	
Yes	99 (16.6)	44 (18.0)	55 (15.6)	

### Factor analysis

We conducted an initial EFA using responses from exclusively IPV survivors to assess for potential scales. Of the initial 33 items, two items - “it is a woman’s responsibility to avoid getting pregnant” and “my husband or boyfriend would be outraged if I asked him to use a condom.” - failed to load at or above 0.3, and so were excluded from further analysis.

Complete responses for all initial 31 items were available for 160/244 (65.6%) of IPV survivors and 218/352 (61.9%) of non-IPV survivors (see Table 2). Preliminary tests found sufficient intercorrelations between items (Bartlett’s test of sphericity p < 0.0001) and sample adequacy (Kaiser-Meyer-Olkin measure of sampling adequacy = 0.707) in the IPV survivor dataset for factor analysis.

**Table 2:** Descriptive statistics for scale/sub-scale items among survivors and non-survivors*.

Scale/sub-scale item	Survivors						Non-survivors					
	n	Mean (Std. dev)*	Endorsement by category (%)				n	Mean (Std. dev)	Endorsement by category (%)			
			SA	A	D	SD			SA	A	D	SD
My community thinks that if a man has paid *lobola* for his wife, he owns her.	237	2.603 (0.997)	21.50	33.30	29.10	16.10	329	2.356 (0.984)	17.00	21.30	41.90	19.80
My community thinks that children belong to a man and his family.	235	2.57 (0.978)	23.00	23.40	41.30	12.30	343	2.475 (0.954)	18.60	24.50	42.60	14.30
My community thinks that a man should have the final say in all family matters.	232	2.659 (0.921)	21.60	32.30	36.60	9.50	333	2.453 (0.939)	16.50	27.30	41.20	15.00
My community thinks that a woman needs her husband’s permission to do paid work.	225	2.671 (0.901)	18.20	41.80	28.90	11.10	322	2.665 (0.9)	17.70	42.50	28.30	11.50
My community thinks that if a man has paid *lobola* for his wife, she must have sex when he wants it.	228	2.601 (0.995)	22.40	30.20	32.50	14.90	320	2.328 (1.042)	18.10	21.60	35.30	25.00
My community thinks that there is nothing a woman can do if her husband wants to have girlfriends.	232	2.065 (0.858)	8.62	14.22	52.16	25.00	331	1.973 (0.784)	5.74	12.08	55.89	26.28
My community thinks that if a woman works she should give her money to her husband.	215	2.484 (0.932)	17.20	27.40	41.90	13.50	307	2.466 (0.912)	15.00	30.60	40.40	14.00
My community thinks that if a man beats you it shows that he loves you.	234	2.068 (0.836)	7.26	16.67	51.71	24.36	338	1.858 (0.832)	5.62	11.54	45.86	36.98
My community thinks that a woman cannot refuse to have sex with her husband.	228	2.636 (0.95)	20.60	35.10	31.60	12.70	312	2.663 (0.955)	22.40	33.30	32.40	11.90
My community thinks that if a wife does something wrong her husband has the right to punish her.	233	2.348 (0.958)	13.30	29.18	36.48	21.03	335	2.227 (0.884)	8.66	26.87	42.99	21.49
My community thinks that a woman should obey her husband.	230	3.348 (0.681)	43.91	49.57	3.91	2.61	337	3.214 (0.773)	38.28	49.26	8.01	4.45
I think that if a wife does something wrong her husband has the right to punish her.	240	1.929 (0.95)	9.10	14.20	37.10	39.60	350	1.889 (0.881)	5.40	17.40	37.70	39.50
I think that if a man has paid *lobola* for his wife, she must have sex when he wants it.	240	1.908 (0.892)	7.50	12.90	42.50	37.10	345	1.846 (0.941)	8.70	11.60	35.40	44.30
I think that if a man beats you it shows that he loves you.	241	1.639 (0.718)	2.90	5.40	44.40	47.30	352	1.46 (0.63)	1.40	3.10	35.50	60.00
I think that if a man has paid *lobola* for his wife, he owns her.	241	2.054 (0.958)	10.80	16.20	40.60	32.40	342	1.865 (0.956)	9.90	9.90	36.90	43.30
I think that a woman needs her husband’s permission to do paid work.	240	2.325 (0.978)	14.20	26.70	36.60	22.50	345	2.391 (0.989)	15.10	30.70	32.50	21.70
I think that if a woman works she should give her money to her husband.	238	2.277 (0.918)	10.92	26.89	41.18	21.01	345	2.232 (0.967)	10.14	30.43	31.88	27.54
There are times when a woman deserves to be beaten.	242	1.715 (0.787)	2.90	12.00	38.80	46.30	349	1.633 (0.764)	2.90	8.90	37.00	51.20
I think that a woman cannot refuse to have sex with her husband.	239	2.209 (0.916)	9.20	26.80	39.70	24.30	335	2.293 (1.02)	16.70	20.60	37.90	24.80
I think that a woman should obey her husband.	242	3.087 (0.787)	31.40	50.00	14.50	4.10	349	2.926 (0.91)	27.50	47.90	14.30	10.30
I think that there is nothing a woman can do if her husband wants to have girlfriends.	241	1.647 (0.756)	2.90	8.30	39.42	49.38	347	1.556 (0.700)	2.31	5.19	38.33	54.18
I think that a man should have the final say in all family matters.	240	2.179 (0.913)	10.42	20.83	45.00	23.75	350	2.066 (0.929)	10.29	15.71	44.29	29.71
I think that children belong to a man and his family.	242	2.120 (0.914)	11.16	14.88	48.76	25.21	352	1.966 (0.936)	10.23	11.36	43.18	35.23
My community thinks that in some rape cases women actually want it to happen.	231	1.983 (0.839)	4.80	19.90	44.10	31.20	327	1.924 (0.793)	5.50	11.30	53.20	30.00
My community thinks that when a woman is raped, she is usually to blame for putting herself in that situation.	234	1.962 (0.881)	8.10	12.40	47.00	32.50	333	1.886 (0.864)	6.60	12.30	44.20	36.90
My community thinks that in any rape case one would have to question whether the victim is promiscuous.	220	2.141 (0.903)	8.70	22.70	42.70	25.90	312	2.003 (0.895)	9.00	13.10	47.10	30.80
My community thinks that if a woman doesn’t physically fight back, it’s not rape.	235	2.038 (0.859)	8.09	14.47	50.64	26.81	329	1.985 (0.857)	6.38	16.72	45.90	31.00
I think that in some rape cases women actually want it to happen.	243	1.724 (0.809)	4.10	10.30	39.50	46.10	344	1.622 (0.722)	2.60	6.40	41.60	49.40
I think that when a woman is raped, she is usually to blame for putting herself in that situation.	242	1.574 (0.673)	2.10	4.10	43.00	50.80	344	1.532 (0.716)	2.90	4.40	35.70	57.00
I think that in any rape case one would have to question whether the victim is promiscuous.	235	1.86 (0.892)	6.80	13.20	39.20	40.80	332	1.729 (0.875)	6.00	10.20	34.40	49.40
I think that if a woman doesn’t physically fight back, it’s not rape.	241	1.697 (0.772)	4.15	6.64	43.98	45.23	341	1.704 (0.842)	5.87	7.33	38.12	48.68

*Table shows responses to each item statement according to a four-point scale: 1 = strongly disagree, 2 = disagree, 3 = agree, 4 = strongly agree.

Scree test and Velicer’s MAP test both suggested limiting extraction to three factors, collectively accounting for 61% of the variance among responses to the items (Factor 1: eigenvalue = 7.27; proportion = 0.35; Factor 2: eigenvalue = 3.29; proportion = 0.16; Factor 3: eigenvalue = 2.32; proportion = 0.11).

One set of 11 items clustered around respondent perceptions of **Community norms about male dominance over female intimate partners (C-MDP)**. This set included perceived norms in the community that men were entitled to assert exclusive control of women in intimate partnerships – ownership of, control over, and right to punish his wife and children; control over the timing of sex; right to have other intimate partners, and control over whether a woman can undertake paid work and the money she earns from that work. For example, one C-MDP item is: “My community thinks that if a man has paid *lobola* for his wife, he owns her.” The set also included statements of community norms that endorsed beliefs that through beating a woman a man shows love for her; and that a woman should obey her husband. The scale of items had similar high internal consistencies among survivors (McDonald’s Omega ≥ 0.86) & non-survivors (McDonald’s Omega ≥ 0.85) (see Table 3).

**Table 3:** Factor loadings* and internal consistency (last row) for community norms about male dominance over female partner (C-MDP), among survivors and non-survivors.

Item	Among survivors (n = 160)			Among non-survivors (n = 218)		
	Community norms endorsing male dominance over female partner (C-MDP)	Individual beliefs endorsing male dominance over female partner (I-MDP)	Survivor culpability for IPV experienced (SBA)	Community norms endorsing male dominance over female partner (C-MDP)	Individual beliefs endorsing male dominance over female partner (I-MDP)	Survivor culpability for IPV experienced (SBA)
My community thinks that if a man has paid *lobola* for his wife, he owns her	**0.7245**	0.0097	0.1341	**0.7014**	0.0846	−0.0314
My community thinks that children belong to a man and his family.	**0.6901**	−0.1271	0.041	**0.5956**	0.1022	−0.0708
My community thinks that a man should have the final say in all family matters.	**0.6092**	−0.0257	0.1437	**0.5838**	0.245	0.0163
My community thinks that a woman needs her husband’s permission to do paid work.	**0.6084**	0.1902	−0.0327	**0.3664**	0.3506	−0.0262
My community thinks that if a man has paid *lobola* for his wife, she must have sex when he wants it	**0.6045**	0.0581	0.2077	**0.7506**	0.0606	0.0073
My community thinks that there is nothing a woman can do if her husband wants to have girlfriends.	**0.5542**	−0.099	0.4034	**0.5632**	0.0079	0.0402
My community thinks that if a woman works she should give her money to her husband.	**0.5336**	0.2086	−0.0714	**0.0946**	0.5771	−0.1328
My community thinks that if a man beats you it shows that he loves you.	**0.4939**	−0.0046	0.4124	**0.6488**	0.0139	0.0971
My community thinks that a woman cannot refuse to have sex with her husband.	**0.4824**	0.1157	0.1494	**0.4824**	0.0834	−0.2026
My community thinks that if a wife does something wrong her husband has the right to punish her.	**0.4653**	0.2317	0.2789	**0.6312**	0.1179	0.0348
My community thinks that a woman should obey her husband.	**0.389**	0.0649	−0.0359	**0.2636**	0.3664	−0.1782
**Internal consistency reliability (ω)**	**0.8594**			**0.8535**		

*Exploratory factor analysis specifying 3 factors and oblique rotation; internal consistency reliability (McDonald’s omega (ω)) for each sub-scale based on items in bold.

Another set of 12 items clustered around **Individual beliefs about male dominance over female intimate partners (I-MDP)**. Item statements in this set include the respondent’s own beliefs that: a man has ownership of and control over his wife and children; her husband controls whether a woman can undertake paid work and the money she earns; being beaten by her husband is sometimes deserved, and an act of love; punishment by her husband is acceptable; husbands can have girlfriends without recourse; and, through payment of *lobola*, she is owed by her husband and obligated to have sex when he wants. An example I-MDP item is: “I think that if a man beats you it shows that he loves you.” IPV survivors’ agreement with these statements appear consistent with an internalised belief that male dominance and IPV is accepted as a part of their role or station in marriage, also described as characterological self-blame [5,7]. The scale of items had similar high internal consistencies among survivors (McDonald’s Omega ≥ 0.83) & non-survivors (McDonald’s Omega ≥ 0.84) [5,7] (see Table 4).

**Table 4:** Factor loadings* and internal consistency (last row) for Individual beliefs about male dominance over female partner (I-MDP), among survivors and non-survivors.

Item	Among survivors (n = 160)			Among non-survivors (n = 218)		
	Community norms endorsing male dominance over female partner (C-MDP)	Individual beliefs endorsing male dominance over female partner (I-MDP)	Survivor culpability for IPV experienced (SBA)	Community norms endorsing male dominance over female partner (C-MDP)	Individual beliefs endorsing male dominance over female partner (I-MDP)	Survivor culpability for IPV experienced (SBA)
I think that if a wife does something wrong her husband has the right to punish her.	−0.0737	**0.7443**	0.0592	0.0741	**0.5637**	0.1508
I think that if a man has paid *lobola* for his wife, she must have sex when he wants it.	0.0207	**0.6638**	−0.0364	0.1751	**0.5572**	0.1619
I think that if a man beats you it shows that he loves you.	−0.1043	**0.614**	0.1343	0.1044	**0.3945**	0.2748
I think that if a man has paid *lobola* for his wife, he owns her.	0.1659	**0.6106**	−0.0985	0.0927	**0.6351**	0.0648
I think that a woman needs her husband’s permission to do paid work.	0.1749	**0.6002**	−0.2056	−0.0851	**0.6638**	0.0169
I think that if a woman works she should give her money to her husband.	0.2701	**0.5403**	−0.248	−0.0466	**0.714**	−0.0811
There are times when a woman deserves to be beaten.	−0.1522	**0.5004**	0.0921	−0.0657	**0.2249**	0.1436
I think that a woman cannot refuse to have sex with her husband.	0.1128	**0.485**	−0.0445	0.0092	**0.5158**	−0.1548
I think that a woman should obey her husband.	0.074	**0.4271**	−0.2461	0.0409	**0.5657**	−0.0674
I think that there is nothing a woman can do if her husband wants to have girlfriends.	0.0274	**0.4246**	0.1904	0.0145	**0.3625**	0.2828
I think that a man should have the final say in all family matters	0.2772	**0.4118**	−0.0278	0.1436	**0.6164**	0.0835
I think that children belong to a man and his family.	0.2851	**0.326**	−0.0884	0.1482	**0.5282**	0.032
**Internal consistency reliability (ω)**		**0.8322**			**0.8373**	

*Exploratory factor analysis specifying 3 factors and oblique rotation; internal consistency reliability (McDonald’s omega (w)) for each subscale based on items in bold.

A third set of eight (8) items clustered around beliefs about **Survivor blaming attitudes (SBA)** towards IPV. This set of individual and community beliefs cluster around the notion that it was a survivor’s behaviour/fault for the physical or sexual violence perpetrated by a man towards his female partner. The statement items include both community norms and individual respondent beliefs that: some rape survivors wanted the rape to occur; the survivor is somehow to blame for putting herself in the situation (including being promiscuous); and she would not have been raped had she resisted physically. Among respondents who had survived IPV, these attitudes reflect both an expectation of how communities might react to a woman who has survived IPV (***anticipated IPV stigma)*** and individual self-blame (***internalised IPV stigma***) for the forced sex they experienced. See Table 5 for details on individual item loadings and internal consistency between the correlated items. The scale items had slightly higher internal consistencies among survivors (McDonald’s Omega ≥ 0.83) compared to non-survivors (McDonald’s Omega ≥ 0.82) (see Tables 3–5).

**Table 5:** Factor loadings* and internal consistency (last row) for survivor blaming attitudes (SBA), among survivors and non-survivors.

Item	Among survivors (n = 160)			Among non-survivors (n = 218)		
	Community norms endorsing male dominance over female partner (C-MDP)	Individual beliefs endorsing male dominance over female partner (I-MDP)	Survivor culpability for IPV experienced (SBA)	Community norms endorsing male dominance over female partner (C-MDP)	Individual beliefs endorsing male dominance over female partner (I-MDP)	Survivor culpability for IPV experienced (SBA)
My community thinks that in some rape cases women actually want it to happen.	0.2801	−0.0792	**0.7322**	0.4219	−0.2122	**0.5443**
My community thinks that when a woman is raped, she is usually to blame for putting herself in that situation.	0.3281	−0.1679	**0.7118**	0.3234	−0.2164	**0.5118**
My community thinks that in any rape case one would have to question whether the victim is promiscuous.	0.0789	−0.206	**0.7116**	0.2268	−0.1713	**0.4574**
My community thinks that if a woman doesn’t physically fight back, it’s not rape.	0.3328	−0.0678	**0.6269**	0.2646	−0.1972	**0.6737**
I think that in some rape cases women actually want it to happen.	−0.1243	0.283	**0.5837**	−0.0066	0.1886	**0.6292**
I think that when a woman is raped, she is usually to blame for putting herself in that situation.	−0.0904	0.2368	**0.5545**	−0.1845	0.2073	**0.7264**
I think that in any rape case one would have to question whether the victim is promiscuous.	−0.3009	0.2004	**0.547**	−0.1273	0.1761	**0.4724**
I think that if a woman doesn’t physically fight back, it’s not rape (norapei)	−0.1337	0.4559	**0.3939**	−0.205	0.1755	**0.7343**
**Internal consistency reliability (ω)**			**0.8282**			**0.8150**

*Exploratory factor analysis specifying 3 factors and oblique rotation; internal consistency reliability (McDonald’s omega (w)) for each subscale based on items in bold.

### Cross-loadings

A number of items cross-loaded on several factors. Four items cross-loaded on Survivor blaming attitudes (SBA) and Community norms about male dominance over female intimate partners (C-MDP) factors. A link between the transgression of normative beliefs of male dominance over their female partners and attributing blame to IPV survivors for the violence they experienced seems plausible. These cross-loadings were resolved as follows:

- Responses to ‘My community thinks that there is nothing a woman can do if her husband wants to have girlfriends’ was dropped from the Survivor blaming attitudes (SBA) scale. Semantically, the item concerns community perceptions of male dominant behaviour than the stigma a survivor might face as the item loaded more strongly on Community norms about male dominance over female intimate partners (C-MDP) factors (0.5542 vs 0.4034). Semantically, the attitude also appears more related to male dominance over their partner compared with self-blame related to IPV survivorship.
- Responses to ‘My community thinks that if a man beats you it shows that he loves you’ showed a slightly stronger loading on Community norms about male dominance over female intimate partners (C-MDP) compared with Survivor blaming attitudes (SBA) (0.4939 vs. 0.4124), and subsequently dropped from the latter.
- Responses to ‘My community thinks that if a woman doesn’t physically fight back, it’s not rape’ was dropped from the Community norms about male dominance over female intimate partners (C-MDP) scale, as it loaded more strongly on the Survivor blaming attitudes (SBA) factor (0.3328 vs. 0.6269). From a survivor perspective, it may suggest self-blame.
- Responses to ‘My community thinks that when a woman is raped, she is usually to blame for putting herself in that situation’ was dropped from the Community norms about male dominance over female intimate partners (C-MDP) scale, as loaded was stronger on the Survivor blaming attitudes (SBA) factor (0.3281 vs. 0.7118). From a survivor perspective, it may suggest self-blame.

Responses to ‘I think that if a woman doesn’t physically fight back, it’s not rape’ loaded both on the construct of male dominance over female intimate partners (I-MDP) and survivor blaming attitudes (SBA) (0.4559 vs. 0.3939, respectively). Although it loaded more strongly on the former (I-MDP), the item appeared semantically more consistent with other SBA items; it was subsequently dropped from I-MDP and retained only on the SBA scale.

### Construct validity

Concerning known group validity, we hypothesised that stigma would be more salient and felt more strongly among IPV survivors as compared with non-IPV survivors (H1). We found median scores higher for all three scales among IPV survivors compared with non-survivors, albeit significantly higher only for the C-MDP scale (IPV survivors: median 28.0 (IQR 24–32), compared with IPV non-survivors: median 26 (IQR 23–31); p = 0.03) (see Table 6).

**Table 6:** Construct validity of identified scales in relation to associated psychosocial outcomes.

	Community norms about male dominance over female partner			Individual beliefs about male dominance over female partner			Survivor blaming attitudes		
	Survivors	Non-survivors	p-value	Survivors	Non-survivors	p-value	Survivors	Non-survivors	p-value
Overall median score									
n	171	241	0.03	221	314	0.08	209	286	0.10
Median (IQR)	28 (24–32)	26 (23–31)		25 (21–29)	24 (20–28)		16 (12–18)	14 (11–16)	
By psychosocial outcomes*									
Attempted suicide?									
No*	27 (24–31)	26 (22–30)		25 (21–29)	24 (19–27)		15 (12–16)	14 (11–16)	
Yes*	29 (26–38)	27.5 (25.5–32)		25 (21–30)	23 (20–28)		17 (10–19)	15.5 (11–18)	
	p = 0.04	p = 0.13		p = 0.80	p = 0.42		p = 0.07	p = 0.14	
Thought about ending life in past month?									
No*	28 (24–32)	26 (22–31)		25 (21–29)	24 (19–28)		15 (12–17)	14 (11–16)	
Yes*	28.5 (26–36)	30 (25–32)		25 (21–27)	24.5 (21–27.5)		16 (15–19)	16 (12–18)	
	p = 0.07	p = 0.18		p = 0.80	p = 0.63		p = 0.02	p = 0.15	
Disclosed abuse to someone?									
No*	29 (26–33)			25 (21–30)			15 (12–18)		
Yes*	26 (23–30)			25 (21–29)			16 (12–17)		
	p = 0.002			p = 0.77			p = 0.75		
Depressive symptoms (CES-D)**	0.0291	0.143	0.02***	0.036	0.137	0.04	0.063	0.010	0.43
	p = 0.71	p = 0.03		p = 0.59	p = 0.01		p = 0.37	p = 0.86	
At risk of clinical depression (CES-D≥16)									
No*	28 (23.5–31.5)	26 (22–30)		25 (20–29)	24 (19–27)		15 (11–16)	14 (11–16)	
Yes*	28 (24–32)	27 (24–32)		25 (21–30)	25 (22–28)		16 (12–19)	14 (12–17)	
	p = 0.71	p = 0.20		p = 0.42	p = 0.06		p = 0.10	p = 0.84	
Post-traumatic stress (HTQ)**	0.118	0.104	0.17	0.014	0.056	0.01	0.135	0.092	0.38
	p = 0.13	p = 0.13		p = 0.84	p = 0.35		p = 0.06	p = 0.14	
At risk of PTSD (Avg HTQ raw score ≥2.5)									
No*	28 (24–32)	26 (22.5–30)		25 (21–29)	24 (19–27)		15 (12–17)	14 (11–16)	
Yes*	28 (25.5–34)	29 (25–32)		24 (19–30)	27 (22–31)		16 (12–18)	16 (14–17.5)	
	p = 0.37	p = 0.06		p = 0.43	p = 0.006		p = 0.32	p = 0.01	

*Median and IQR, P-values obtained from t-test if distribution parametric, or Wilcoxon rank-sum test if distribution non-parametric.

**Spearman’s rank correlation coefficient (ρ) and significance test of independence.

***P-value estimate presented is interaction term of regression analysis.

Concerning community norms favouring male power over their partner, among survivors, higher C-MDP scale scores were associated with disclosures of having attempted suicide in the past (p = 0.04) and having thought about ending life in the past month (p = 0.07) (H2-H3).

Survivors who had disclosed to someone they had experienced abuse, had lower C-MDP scale scores compared with survivors who had not disclosed (p = 0.002) (H10). That is, survivors who more strongly endorsed statements suggesting male dominance over female partners were normal in their community were less likely to have disclosed abuse to someone, compared with survivors who endorsed such statements less strongly.

Among non-survivors, higher C-MDP scores were also found among respondents who disclosed having attempted suicide (p = 0.13) (H6). Among these respondents, stronger endorsement of community norms that favour male dominance over their partners also correlated with stronger depressive symptoms (p = 0.03) (H8). Our analysis, however, did not find the proportion of non-survivors at risk of clinical depression varied significantly by C-MDP score (p = 0.2) (see Table 6).

Regarding individual beliefs favouring male power over their partner, we did not detect significant associations between I-MDP score and psychosocial outcomes among IPV survivors. Among non-IPV survivors, greater I-MDP scores correlated with stronger depressive symptoms (p = 0.01) and risk of PTSD (p = 0.006) (H8). We found risk of clinical depression varied only marginally by I-MDP score (p = 0.06).

Among IPV survivors, endorsement of survivor blaming attitudes (SBA) correlated with disclosure of having attempted suicide (p = 0.07) and having thoughts about ending life in the past month (p = 0.02) (H2-H3). Among survivors, greater endorsement of SBA also correlated with symptoms of post-traumatic stress (p = 0.06) (H5), however endorsement of SBA did not vary significantly with disclosing abuse to others (p = 0.75). Among non-IPV survivors, we found greater SBA endorsement correlated with risk of PTSD (p = 0.01).

## Discussion

This paper aims to identify and explore the psychometric properties of scales for gender beliefs and norms, and to explore their relationship with anticipated and internalised stigma among women survivors of IPV living in Botswana. We found good internal consistency among items within each of the three scales (C-MDP (11 items); I-MDP (12 items); SBA (8 items)) identified through the exploratory factor analysis among both survivors and non-survivors of IPV.

Scale items were reconfigured slightly for five cross-loaded items. Two items were excluded from SBA because they loaded slightly more strongly on C-MDP; the items were also statements concerning perceived community norms of more general male dominance, rather than survivor blaming attitudes. Two other items specifically concerning culpability of survivors also loaded more strongly on SBA, and so were excluded from C-MDP. One cross-loaded item concerning survivor culpability was deemed semantically aligned with other SBA scale items and dropped from I-MDP.

### Differences in factor loadings between survivor and non-survivors

While comparing factor loadings for differences between IPV-survivors and non-survivors, we noted items of several scales that loaded strongly *only for IPV-survivors* (that is, scale item with factor loading >0.3 among IPV survivors and <0.3 among non-survivors).

Concerning the C-MDP scale, two items loaded strongly on the construct among respondent perceptions of IPV survivors alone: (i) my community thinks that if a woman works, she should give her money to her husband (IPV: 0.554 vs. non-IPV: 0.095); and (ii) my community thinks that a woman should obey her husband (IPV: 0.389 vs. non-IPV: 0.264). Such sentiments were once supported by common law in Botswana. Prior to 2004, married women did not have the right to own property, and husbands were recognised as the *de facto* head of their family [53]. Although this changed with the introduction of the Abolition of Marital Powers Act (2004), customary or religious marriages remain unbound by common law [54]. The inclusion of financial control in the context of violence is consistent with findings of Modie-Moroka, who described the expectations within Botswana communities for men to be the heads of the households and to control its financial assets and other resources. She noted that violence often supports the assertion of authority to do so [55]. Although the notion that women should give their money to their husbands may seem contradictory to patriarchal roles that ascribe men as providers [56], women may persist in relationships not only for fear of retaliation from leaving a violent intimate partner [57], but the stigma associated with losing status. Malinga explains that for women in Botswana, marriage implies “womanhood, self-definition, and gain in social empowerment and social status.” Among low-income, unmarried women in violent relationships, even the prospect of getting married in the future was adequate motivation to stay in a relationship with a violent partner [58].

In comparing factor loadings of individual beliefs of male dominance over their partners (I-MDP), one item exclusively loaded strongly on I-MDP among survivors and not among non-survivors: there are times when a woman deserves to be beaten (IPV: 0.500 vs. non-IPV: 0.225). From a survivor’s perspective, endorsement may suggest some personal doubt as to whether their IPV experience was wrong and that violence against them could be justified and even deserved. Such internalised stigma [2], particularly behavioural self-blame [7], uniquely forms part of the individual beliefs of male dominance over intimate partners for survivors of abuse.

Nearly half of all women endorsed at least one of the eight SBA items (described in detail elsewhere [24]). Comparing survivor and non-survivor responses, all SBA items loaded with similar strength (>0.3) for all eight items. We noted relatively few differences in loadings between survivors and non-survivors concerning Survivor blaming attitudes (SBA). This suggests the underlying structure for SBA items is consistent and does not vary with personal experience of IPV. The SBA items concern behavioural self-blame [7], likely formed and shaped through women’s exposure to general societal stigma towards female survivors [5]. As the items that loaded on the SBA factor are phrased to describe general rape and self-blame, respondents also may have considered situations involving non-partner rape when rating their level of endorsement for each of its items.

### Comparison with other stigma measures intended for IPV

Aspects of Murray, Crowe, and Overstreet’s model for IPV stigmatisation were reflected in two of the three scales. SBA appears to elicit cultural (e.g., ‘I think that in some rape cases women actually want it to happen.’, ‘I think that if a woman doesn’t physically fight back, it’s not rape’) and anticipated (e.g., ‘My community thinks that in any rape case one would have to question whether the victim is promiscuous.’) sources of stigma [2]. I-MDP items appear to reflect a combination of IPV stigma sources – cultural (e.g., ‘There are times when a woman deserves to be beaten.’), perpetrator (e.g., as implied by an IPV survivor’s endorsement of ‘I think that if a man beats you it shows that he loves you.’), and internalised (e.g., as implied by an IPV survivor’s endorsement of ‘I think that if a wife does something wrong her husband has the right to punish her.’) – as well as statements consistent with endorsement of internalised gender inequity (e.g., ‘I think that a man should have the final say in all family matters’). In contrast, C-MDP items, appear to reflect perceptions of gender-inequitable community norms that legitimize IPV. Endorsement of the items imply a perception that community norms of male dominance over female partners are prominent in each respondent’s reference community; however, unlike existing scales for IPV stigma among survivors [59,60], none of the items of this study’s three scales directly assess anticipated or enacted IPV stigma. Although absent from existing IPV survivor stigma scales, the concept of *lobola/bogadi* and its perceived implications for sexual decision-making, control and violence, featured prominently in both the male dominance over partner scales. Consistent with Yang’s WMM theory, integrating cultural practices like the concept of *lobola/bogadi* within scale items may strengthen the sensitivity of IPV stigma scales intended for this population [18]. We recommend further research to identify and explore other culturally influenced social norms relevant for stigma experienced by heterosexual female IPV survivors.

Of the three scales, only scores for C-MDP were significantly higher among survivors compared with non-survivors, suggesting community normative beliefs of male dominance over their female partners were particularly salient for survivors. As well, IPV stigma measures incorporating such community normative beliefs may elicit stronger responses from survivors, compared with measures that do not.

### Implications for mental health and disclosure

The construct validity analyses yielded important insights about the use of community and individual gender beliefs and survivor stereotyping as proxy measures for IPV stigma.

Affirming statements that a survivor is to blame or is culpable for the violence she experiences from an intimate partner, also may be linked with some psychosocial outcomes. This study observed a potential association between endorsement of survivor blaming attitudes (SBA) and mental health measures for post-traumatic stress (p = 0.06) and suicidal ideation in the past month (p = 0.02) among IPV survivors. This suggests that holding survivor blaming attitudes may influence mental wellbeing and sense of hopelessness amidst their anticipation of shame by others. The finding is consistent with those of Nöthling et al. [48] who also reported higher PTSD scores among survivors experiencing higher levels of rape stigma. Understanding the severity of internalised stigma experienced holds relevance for the management of post-traumatic stress among such survivors of IPV in Botswana. Overcoming stigma is a recognised component of the recovery process for survivors of abuse [61]. The survivor’s acknowledgement and understanding of self-blame, social devaluation, and discrediting forms part of the intrapersonal processes of recovering from IPV [62].

For both C-MDP and I-MDP, among non-survivors, we observed higher median scores associated with greater depressive symptoms. This finding may have negligible clinical importance, since neither scale’s median score difference coincided with a statistically significant difference in risk of clinical depression (i.e. CES-D score ≥16). Nonetheless, endorsement of scale items reflects an acknowledgement of individual and perceived community norms of an important form of gender discrimination. In the absence of a strong predictor of depression, like physical or sexual IPV, gender discrimination may independently impede healthy lifestyles that support mental health. Such findings have been reported among women in Botswana and other settings [63–65].

Among non-survivors, median scores of all three scales appeared higher among participants at risk of PTSD (i.e., average HTQ raw score ≥2.5); this difference was statistically significant for both I-MDP and SBA scales. Such women may have experienced forms of trauma unrelated to physical or sexual IPV (e.g., through experiences of non-partner rape, emotional intimate partner violence, childhood trauma), with their levels of post-traumatic stress exacerbated by exposures that also increase with I-MDP and SBA endorsement. We recommend further research in this area.

A key finding of this nationally representative study concerned C-MDP endorsement and disclosure of abuse. Among women who have experienced physical and/or sexual IPV, our quantitative analysis suggests that a woman who believes her community accepts patriarchal norms is less likely to disclose her abuse to someone, compared with one who does not believe this. This association is independent of their individual beliefs about those norms (I-MDP), their endorsement of SBA, mental health, age, formal education, employment, or food insecurity. This finding encourages recognition that community-based, primary and secondary IPV prevention efforts to shift societal gender norms may also yield tertiary prevention benefits. It also suggests that, in addition to improving health care, legal and law enforcement service delivery, interventions to improve IPV disclosure and help-seeking by women survivors need to focus beyond service delivery to be effective at a population level.

### Limitations

This secondary analysis of has several limitations. The primary study minimised potential selection biases by implementing a multi-stage, community-based, random sample, designed by the national statistics office and implemented by extensively trained fieldworkers [24]. Its response rate is comparable to other national studies [66,67] with findings generalized to Botswana. The authors however acknowledge that women who have died – including those who have died from partner-related violent acts - and homeless and transient women, would not have been included in the sample. As well, those who comprised the 15% of the individuals enumerated in sampled households who did not participate may have responded differently than those included in the sample.

The stigma experienced by survivors of IPV may have contributed to underreporting. Although potential reporting biases were likely minimised through adherence to data collection procedures safeguarding privacy, confidentiality and full participation of participants [24], there is a risk of differential misclassification of survivors who did not report their abuse, as non-survivors. Analyses comparing survivors and non-survivors may bias results towards the null, and results of mental health analyses restricted to non-survivor groups may yield results more similar to those done with survivor groups.

We discussed differences in item loadings across sampled subgroups under an assumption that IPV survivors had a different conceptual frame of reference for the factors compared with non-survivors, arising from plausible experiential or contextual differences related to IPV survivorship. Such intergroup differences in factor loadings may also have arisen from differences in group size or even the characteristics of participants randomly sampled from each group. Further application of these measures in other studies will help confirm if their psychometric properties are reproducible. Ideally, future confirmatory factor analyses should involve samples able to accommodate 5–10 participant observations per indicator variable study data of sample size [68].

All exploratory construct validity analyses are bivariate. Further exploration using multivariate and advanced statistical techniques may be possible in future studies with larger sample sizes. Scale items were also administered at one time point, precluding the opportunity to assess test-retest reliability. The cross-sectional study design also limited our ability to ascertain if the salience of these attitudes preceded or followed participant experience of IPV.

We could not explore concurrent validity with other IPV stigma scales, since they were not administered. Future GBV studies should be encouraged to administer IPV stigma scales.

To ascertain the influence of missing data, we conducted sensitivity analyses by re-running our analyses on datasets completed by replacing missing responses with mean imputed values. Those analyses yielded similar results in terms of the nature and composition of extracted factors, known group validity, and convergent validity. See S1 File for details of these analyses.

We did not include the following statement item from the community ideas about rape scale measures: “it is possible for a woman to be raped by her husband.” Since marital rape was not recognised as an offense under either the Botswana Penal Code [23] or Domestic Violence Act [69] (and remained not recognised by laws at the time of submission of this manuscript), it is difficult to determine if disagreement with the statement reflects a genuine belief that being married implies ‘perpetual consent to sex’ – that is, women cannot be raped by their husbands because they believe they consented to have sex whenever their husband wanted as part of their commitment to marriage, or internalised stigma in the form of self-blame. This extends further to the concern of seeking help, since women who suffered violence from their husbands may find themselves less empowered to do so if it is not recognised as an illegal act by Botswana’s laws. Future studies should consider the impact of the absence of penal code recognition of marital rape on stigma to IPV.

## Conclusions

This study presents three psychometrically tested scales for measuring norms and beliefs about power dynamics and violence in heterosexual relationships in Botswana. Women IPV survivors who endorse such scales are likely to experience more intense forms of anticipated, enacted and internalised IPV stigma. We found that women IPV survivors who perceived strong community endorsement of violent, patriarchal social norms were more likely to have contemplated or attempted suicide, and less likely to have disclosed their abuse to someone. In contrast, among non-IPV survivors, women who perceived strong community endorsement of violent, patriarchal social norms were more likely to have depressive symptoms, as were those who themselves accepted violent, patriarchal intimate partnerships as normal. Holding survivor blaming attitudes correlated with contemplating suicide among IPV survivors, and while among non-IPV survivors holding such attitudes correlated with risk of PTSD.

Overall, the findings support the importance of gendered social norms in understanding how survivors experience IPV stigma. We recommend structural equation modelling to test these relationships. Future research to identify other culture-informed practices like giving *lobola/bogadi*, that shape IPV survivors’ experiences of stigma. Mechanisms for socialising beliefs around male dominance over their partners should be explored as targets for future interventions to improve IPV disclosure. Finally, these secondary analyses utilized items of several scales that are commonly included in GBV studies in other countries. Such analyses may be considered with data from other southern African settings where similar gendered cultural norms prevail.

## Supporting information

S1 Checklist Inclusivity in global research questionnaire. (DOCX)

S1 Data All data for the 596 participants analysed for this report. (XLS)

S1 File Sensitivity analysis assessing the influence of missing data. (DOCX)
