# Supplementary material for: Gender beliefs and norms underlying intimate partner violence stigma among women living in Botswana: Results of an exploratory factor analysis
Source: PLOS Glob Public Health. 2025 Feb 5;5(2):e0004113. doi: 10.1371/journal.pgph.0004113 (PMC11798532; doi:10.1371/journal.pgph.0004113)
Supplement: S1 Checklist — (DOCX) [file pgph.0004113.s001.docx]

Inclusivity in global research

PLOS’ policy on inclusivity in global research aims to improve transparency in the reporting of research performed outside of researchers’ own country or community and ensures that PLOS publications reporting global research adhere to high standards for research ethics and authorship. Authors of relevant research articles may be asked to complete the questionnaire below, which outlines ethical, cultural, and scientific considerations specific to inclusivity in global research. This questionnaire may be requested when researchers have travelled to a different country to conduct research, if research uses samples collected in another country, research with Indigenous populations or their lands, or if research is on cultural artefacts. Researchers travelling to another country solely to use laboratory equipment will not normally be required to complete the questionnaire. However, the questionnaire can be requested at the journal’s discretion for any submission – if you have been requested to complete this questionnaire by the PLOS journal you submitted to, please do so.

Please complete the questionnaire below and include this as a Supporting Information file with your manuscript. Note that if your paper is accepted for publication, this checklist will be published with your article in the supporting information files. Please ensure that you reference the checklist in the main body of your manuscript. We suggest adding a subsection ‘Inclusivity in global research’ to your Methods section and adding the following sentence: “Additional information regarding the ethical, cultural, and scientific considerations specific to inclusivity in global research is included in the Supporting Information (SX Checklist)”

The questions have been designed to be applicable to a wide range of study types, and there are subsections for both human subjects research and non-human subjects research. If any of the questions are not relevant to your research please mark them as “N/A” as appropriate.

**Ethical considerations, permits and authorship**

*This section is applicable to all research types.*

Provide details as to who granted permissions and/or consent for the study to take place in the Methods section of your manuscript. This should include the names of **all** ethics boards, governmental organizations, community leaders or other bodies that provided approval for the study. If individuals provided approval refer to these people by their role or title but do not list their name(s).

**Reported on page number: 7**

**Permission to carry out this study was obtained from the Institutional Review Boards of the University of Botswana (UBR/RES/IRB/BIO/342), the University of the Witwatersrand (M230227 MED23-01-053), and the Government of Botswana (MYSC 9/1/1).** **Primary data collection was approved by the Botswana Ministry of Health Research and Development Division (PPMEE 13/18/ 1 PS IV (37));** permission to access community research sites was obtained through a process of community mobilisation led by the Government of Botswana Woman Affairs Department, described elsewhere **[1];**

If there were any deviations from the study protocol after approval was obtained please provide details of these changes in the Methods section of your manuscript.

**Reported on page number: N/A**

**N/A – concerning this secondary analysis, no deviations from the study protocol after approval were obtained.**

Did this study involve local collaborators that are residents of the country where the research was conducted or members of the community studied? If you do not have any authors from said communities, please provide an explanation for this below.

**Yes. The lead author of this paper is based on secondary analysis of data. The primary data collection occurred through a collaboration between government and a local NGO, following a multi-stage consultation , is a resident of Botswana who has lived and worked in the country since 2007. Co-author Lorato Moalusi is a resident of Botswana. As the study concern data collected through a nationally representative sample, she is ‘from said community’. The other co-authors, Prof Nicola Christofides and Dr Mercilene Machisa, are residents of the neighbouring nation of South Africa.**

Everyone listed as an author should meet PLOS’ criteria for authorship and all individuals who meet these criteria should be included in the author byline, rather than the acknowledgements. For further information please see the journal’s Authorship Policy.

**Noted. We believe the authorship of this manuscript complies with the journal’s policy.**

**Human subjects research (e.g. health research, medical research, cross-cultural psychology)**

Did you obtain written informed consent from a representative of the local community or region before the research took place? How did you establish who speaks for the community? Details of written informed consent obtained from study participants should be reported separately in the Methods section of your manuscript.

>>> Did you obtain written informed consent from a representative of the local community or region before the research took place?

**The primary study was planned and implemented through a Goverment – NGO partnership. The Women’s Affairs Department of the Government of Botswana informed representatives of local communities through each study site’s District Commissioner. This is described in detail elsewhere [1].**

>>> How did you establish who speaks for the community?

**The appropriate local community representative to engage prior to community mobilization was determined by Government through consultation with each study site’s District Commissioner and the district office of the Women’s Affairs Department (WAD) of the Government of Botswana prior to carrying out fieldwork. In farming areas, the study sought permission from land owners to access properties to interview farm workers and other residents. This information is described in an already published primary study report [1].**

>>> Details of written informed consent obtained from study participants should be reported separately

**We reported consent processes in the manuscript’s methods section:**

**“The primary study team collected data in accordance with the WHO Ethical and Safety Recommendations for Research on Domestic Violence against Women [2]. Participants provided written informed consent to participate in the study.”**

**This is a secondary analysis, and details concerning informed consent are published in a primary study report [1].**

How did members of the local community provide input on the aims of the research investigation, its methodology, and its anticipated outcome(s)?

**The Government-NGO led primary study was national in focus. A stakeholder reference group was established to provide input on the aims of the research investigation, methods, outcomes. Its membership included: “Botswana Attorney General, Government Women Affairs Department (WAD), Botswana Police Services (BPS), Ministry of Health, Central Statistical Office, University of Botswana Sociology Department, Women and the Law in Southern Africa (WLSA), civil society organisations and other stakeholders” [1] page 26.**

When engaging with the local community, how did you ensure that the informed consent documents and other materials could be understood by local stakeholders?

**Informed consent and data collection instruments were written in simple language in English and in Setswana (commonly spoken local language). All instruments were confirmed as understandable through independent back-translation [1] page 39, and through review by stakeholder representatives who were members of the reference group [1].**

Will the findings of the research be made available in an understandable format to stakeholders in the community where the study was conducted (e.g. via a presentation, summary report, copies of publications, etc.)? Please provide details of how this will be achieved.

**Yes. Preliminary study findings have already been presented to members of intervention stakeholder groups and Public Health and Mental Health practitioners. As well, this secondary data analysis study is one component of a larger study. At the conclusion of that study, a summary report including these findings will be prepared and presented at a multi-sectoral stakeholder workshop, along with other research products, including copies of this manuscript. A press release is planned to coincide with the workshop, directed at community stakeholders through members of the press and local media. This finding dissemination / stakeholder mobilization activities will be supported through a project funded by the Sexual Violence Research Initiative (SVRI).**

**Yes. Permission was obtained from the Ministry of Youth, Gender, Sport and Culture (MYGSC) of the Government of Botswana as well as the Principal Investigator of the primary study, who is also a co-author of the manuscript. Per agreement with MYGSC, a copy of all of the study’s research outputs will be shared with the government ministry prior to ending the study. As indicated, the primary study was co-led through a Government-NGO partnership. Beyond regulatory approval by the Ministry of Health Research and Development Committee (IRB), permission to conduct the study was self-determined by the Government of Botswana, who developed and disseminated the main output of the primary study.**

**Non-human subjects research using specimens/ animals collected as part of the study, or those housed in archival collections. Examples include archaeology, paleontology, botany and zoology.**

Did the permission you obtained from a local authority to perform the study include an agreement on access to outputs and benefit sharing? This may include procedures to enable fair distribution of the benefits and resources arising from the research performed. Please include any details of Prior Informed Consent and Benefit Sharing Agreements obtained. These may be required by field-specific regulations, for example the Convention on Biological Diversity (CBD) and the associated Nagoya Protocol.

If the material used in your study was imported, please A) provide the year it was imported and B) indicate whether permits were obtained to import/export the materials used, C) provide details of any permits obtained. If this information is not available, please indicate this.

**N/A**

If you used archival specimens, please state how the material used in your study was acquired by the institute it is held in and provide details of any permits obtained for the original excavations/ sample collection. If this information is not available, please indicate this.

**This manuscript reports secondary analysis of anonymized data collected by a study led by several of the manuscript’s co-authors. Details of the local institutional review boards that granted permission to perform the study are indicated in the manuscript subsection on Ethics.**

How was the potential cultural significance of the materials collected in your study to local communities considered in your research design? Were Indigenous peoples and/or local researchers and institutions involved with archaeological excavations / collection of specimens? If so, please provide a description of their involvement.

**The primary study implementation involved extensive engagement with local and national stakeholders. Potential cultural significance falls within the scope of concerns considered by the primary study’s stakeholder reference group [1]. All interviewers and research coordinators who participated in primary data collection were local women citizens of Botswana.**

If your manuscript includes photographs of human remains please indicate whether authors obtained permission from descendants or affiliated cultural communities to do so.

**N/A – no photographs are contained in this manuscript.**

1. Machisa M, van Dorp, R Gender Based Violence Indicators Study Botswana. GL Botswana/Ministry of Labour and Home Affairs, Women’s Affairs Department, 2012 2012. Report No.

2. World Health Organization. Putting women first: Ethical and Safety Recommendations for Research on Domestic Violence Against Women. Geneva, Switzerland2001.
