# Supplementary material for: Gender beliefs and norms underlying intimate partner violence stigma among women living in Botswana: Results of an exploratory factor analysis
Source: PLOS Glob Public Health. 2025 Feb 5;5(2):e0004113. doi: 10.1371/journal.pgph.0004113 (PMC11798532; doi:10.1371/journal.pgph.0004113)
Supplement: S1 File — (DOCX) [file pgph.0004113.s003.docx]

# **S1 File: SENSITIVITY ANALYSES TO ASSESS THE INFLUENCE OF MISSING DATA ON THE EXPLORATORY FACTOR ANALYSIS AND PSYCHOMETRIC ANALYSIS**

## Imputation of incomplete data

To assess the influence of missing data, we performed a sensitivity analysis comparing results of the naïve analysis with one involving a dataset ‘completed’ by single imputation. For each of the 31 candidate scale item variables, we imputed values generated from the mean (rounded up to the next whole number) of the known values of the variable. As we hypothesised the interpretation of a majority of the scale items would differ based on prior experience of physical and sexual violence, we estimated mean separately for survivors and non-survivors before replacement under missing completely at random assumptions. (See below for Stata Code).

Based on minimum average partial correlation for number of principal components (MINAP) procedure and Scree plot test, we extracted the first three (3) factors, rotating factor loadings using promax oblique rotation.

## Influence of missing data on exploratory factor analysis

In the analysis of the mean imputed dataset (MID) or ‘completed’ dataset, the items that clustered around the three extracted factors were very similar to those of the naïve analysis with incomplete data: 11 items around Community Norms about Male Dominance over Female Partner (C-MDP), 12 items around Individual Beliefs about Male Dominance over Female Partner (I-MDP), and 8 items around Survivor Blaming Attitudes (SBA). Only one item “I think that if a woman doesn't physically fight back, it's not rape” loaded relatively stronger on SBA than I-MDP in the MI data analysis. Notably, the item was also retained as a SBA item on semantic justifications in the naïve analysis.

## Influence of missing data on psychometric analyses

Known group validity - To assess the sensitivity of known group validity results to missing data, we examined whether missingness influenced if a scale distinguished between IPV survivors and non-survivors. At the alpha=0.05 significance threshold, MID analysis suggests survivors were slightly more likely to endorse C-MDP and SBA compared with non-survivors. See Tables A and B for details.

Convergent validity - In the MID analysis of survivors, C-MDP endorsement appeared significantly higher among those who had not disclosed their abuse to someone prior to the study, as well as those with greater post-traumatic stress; C-MDP endorsement appeared marginally higher among those survivors who disclosed attempting suicide in the past. Differences by disclosure of suicidal thoughts and depressive symptoms did not vary significantly by C-MDP endorsement in the ‘completed’ dataset.

Among non-survivors, MID analysis found C-MDP endorsement higher among participants indicating greater depressive symptoms, and marginally higher among non-survivors with greater post-traumatic stress. Differences in C-MDP endorsement were not statistically significant for non-survivors who had disclosed attempting suicide or suicidal thoughts.

Concerning psychosocial outcomes related to individual beliefs about male dominance over female partners, the ‘completed’ dataset analysis yielded similar findings to the naïve analysis. I-MDP endorsement appeared significantly greater only among non-survivors expressing greater depressive symptoms.

We also observed similar findings in naïve and ‘completed’ data analysis of SBA endorsement. Among survivors, ‘completed’ data analysis found SBA endorsement higher only among those who disclosed having suicidal thoughts in the past month. See Table C.

## **Table A: Factor loadings* (3 factor solution) and internal consistency (last row), among survivors, analysed using mean imputed dataset (n=244)**

| **Item** | **Factor1**  **C-MDP** | **Factor2**  **I-MDP** | **Factor3**  **SBA** |
| --- | --- | --- | --- |
| My community thinks that if a man has paid Lobola for his wife, he owns her | 0.6873 | 0.0537 | 0.0826 |
| My community thinks that if a man has paid Lobola for his wife, she must have sex when he wants it | 0.6613 | 0.0705 | 0.0807 |
| My community thinks that children belong to a man and his family. | 0.6561 | -0.0811 | -0.0721 |
| My community thinks that a man should have the final say in all family matters. | 0.6132 | -0.0017 | 0.0612 |
| My community thinks that there is nothing a woman can do if her husband wants to have girlfriends. | 0.5412 | -0.1371 | 0.3297 |
| My community thinks that if a man beats you it shows that he loves you. | 0.5064 | -0.0015 | 0.3874 |
| My community thinks that a woman needs her husband's permission to do paid work. | 0.4917 | 0.2943 | -0.0872 |
| My community thinks that if a wife does something wrong her husband has the right to punish her. | 0.4846 | 0.2719 | 0.1955 |
| My community thinks that a woman cannot refuse to have sex with her husband. | 0.4612 | 0.0775 | 0.1209 |
| My community thinks that if a woman works she should give her money to her husband. | 0.436 | 0.1962 | -0.0309 |
| My community thinks that a woman should obey her husband. | 0.3745 | 0.0821 | -0.1214 |
| I think that if a wife does something wrong her husband has the right to punish her. | -0.0437 | 0.7188 | 0.0573 |
| I think that if a man has paid Lobola for his wife, she must have sex when he wants it. | 0.052 | 0.6716 | -0.02 |
| I think that if a man has paid Lobola for his wife, he owns her. | 0.1206 | 0.647 | -0.0677 |
| I think that a woman needs her husband's permission to do paid work. | 0.1111 | 0.6322 | -0.2121 |
| I think that if a man beats you it shows that he loves you. | -0.1232 | 0.5823 | 0.2344 |
| There are times when a woman deserves to be beaten. | -0.1289 | 0.5302 | 0.1427 |
| I think that a woman cannot refuse to have sex with her husband. | 0.0304 | 0.5007 | 0.0348 |
| I think that if a woman works she should give her money to her husband. | 0.1557 | 0.4883 | -0.0648 |
| I think that a man should have the final say in all family matters | 0.2384 | 0.4473 | -0.0564 |
| I think that a woman should obey her husband. | 0.0121 | 0.4308 | -0.2363 |
| I think that there is nothing a woman can do if her husband wants to have girlfriends. | 0.0182 | 0.3583 | 0.2971 |
| I think that children belong to a man and his family. | 0.2555 | 0.3207 | -0.1222 |
| My community thinks that in some rape cases women actually want it to happen. | 0.2957 | -0.1323 | 0.6525 |
| My community thinks that in any rape case one would have to question whether the victim is promiscuous. | 0.126 | -0.1524 | 0.6495 |
| My community thinks that when a woman is raped, she is usually to blame for putting herself in that situation. | 0.3555 | -0.1382 | 0.6334 |
| I think that in some rape cases women actually want it to happen. | -0.1243 | 0.2196 | 0.6125 |
| I think that when a woman is raped, she is usually to blame for putting herself in that situation. | -0.0874 | 0.1897 | 0.5814 |
| I think that in any rape case one would have to question whether the victim is promiscuous. | -0.2397 | 0.1417 | 0.5681 |
| My community thinks that if a woman doesn't physically fight back, it's not rape. | 0.3759 | -0.1282 | 0.5416 |
| I think that if a woman doesn't physically fight back, it's not rape (norapei) | -0.0644 | 0.3234 | 0.4064 |
| Internal consistency reliability (ω) | 0.8403 | 0.8246 | 0.8142 |

## **Table B: Factor loadings* (3 factor solution) and internal consistency (last row), among non-survivors, analysed using mean imputed dataset (n=352)**

| **Item** | **Factor1**  **C-MDP** | **Factor2**  **I-MDP** | **Factor3**  **SBA** |
| --- | --- | --- | --- |
| My community thinks that if a man has paid Lobola for his wife, he owns her | 0.0603 | 0.628 | 0.0823 |
| My community thinks that if a man has paid Lobola for his wife, she must have sex when he wants it | 0.0452 | 0.7003 | 0.0905 |
| My community thinks that children belong to a man and his family. | 0.1433 | 0.6159 | -0.1432 |
| My community thinks that a man should have the final say in all family matters. | 0.1707 | 0.5923 | 0.0448 |
| My community thinks that there is nothing a woman can do if her husband wants to have girlfriends. | 0.0477 | 0.4215 | 0.1731 |
| My community thinks that if a man beats you it shows that he loves you. | 0.0351 | 0.5114 | 0.2456 |
| My community thinks that a woman needs her husband's permission to do paid work. | 0.2723 | 0.4801 | -0.1667 |
| My community thinks that if a wife does something wrong her husband has the right to punish her. | 0.2413 | 0.4586 | 0.1595 |
| My community thinks that a woman cannot refuse to have sex with her husband. | 0.1095 | 0.4952 | -0.1272 |
| My community thinks that if a woman works she should give her money to her husband. | 0.4611 | 0.1852 | -0.1149 |
| My community thinks that a woman should obey her husband. | 0.2876 | 0.3377 | -0.2115 |
| I think that if a wife does something wrong her husband has the right to punish her. | 0.6201 | -0.0279 | 0.2671 |
| I think that if a man has paid Lobola for his wife, she must have sex when he wants it. | 0.5662 | 0.1093 | 0.229 |
| I think that if a man has paid Lobola for his wife, he owns her. | 0.5681 | 0.0452 | 0.2108 |
| I think that a woman needs her husband's permission to do paid work. | 0.591 | 0.0226 | -0.1188 |
| I think that if a man beats you it shows that he loves you. | 0.4362 | -0.0179 | 0.41 |
| There are times when a woman deserves to be beaten. | 0.2938 | -0.1083 | 0.2771 |
| I think that a woman cannot refuse to have sex with her husband. | 0.5023 | 0.0446 | -0.1012 |
| I think that if a woman works she should give her money to her husband. | 0.6317 | -0.039 | 0.0019 |
| I think that a man should have the final say in all family matters | 0.5903 | 0.1399 | 0.1113 |
| I think that a woman should obey her husband. | 0.5792 | -0.0028 | -0.0697 |
| I think that there is nothing a woman can do if her husband wants to have girlfriends. | 0.3399 | -0.0811 | 0.4203 |
| I think that children belong to a man and his family. | 0.5035 | 0.2092 | -0.08 |
| My community thinks that in some rape cases women actually want it to happen. | -0.2206 | 0.4755 | 0.3888 |
| My community thinks that in any rape case one would have to question whether the victim is promiscuous. | -0.1915 | 0.3262 | 0.4504 |
| My community thinks that when a woman is raped, she is usually to blame for putting herself in that situation. | -0.2197 | 0.4113 | 0.389 |
| I think that in some rape cases women actually want it to happen. | 0.1977 | 0.0404 | 0.5483 |
| I think that when a woman is raped, she is usually to blame for putting herself in that situation. | 0.1948 | -0.0924 | 0.5874 |
| I think that in any rape case one would have to question whether the victim is promiscuous. | 0.1356 | -0.0047 | 0.4802 |
| My community thinks that if a woman doesn't physically fight back, it's not rape. | -0.206 | 0.3309 | 0.497 |
| I think that if a woman doesn't physically fight back, it's not rape (norapei) | 0.1219 | -0.1254 | 0.5501 |
| Internal consistency reliability (ω) | 0.8334 | 0.8326 | 0.7841 |

## **Table C: Construct validity of identified scales in relation to associated psychosocial outcomes, analysed using mean imputed dataset**

|  | Community norms about male dominance over female partner | | | Individual beliefs about male dominance over female partner | | | Survivor blaming attitudes | | |
| --- | --- | --- | --- | --- | --- | --- | --- | --- | --- |
|  | Survivors | Non-survivors | p-value | Survivors | Non-survivors | p-value | Survivors | Non-survivors | p-value |
| Overall Median score: |  |  |  |  |  |  |  |  |  |
| n | 244 | 352 | 0.002 | 244 | 352 | 0.06 | 244 | 352 | 0.04 |
| median (IQR) | 28 (24-31) | 26 (23-30) |  | 25 (21-29) | 24 (20-27.5) |  | 15 (12-18) | 14 (11-16) |  |
| By Psychosocial outcomes* | | | | | | | | | |
| Attempted suicide? |  |  |  |  |  |  |  |  |  |
| No* | 27.5 (24-31) | 25 (22-30) |  | 25 (21-29) | 24 (19-27) |  | 15 (12-17) | 14 (11-16) |  |
| Yes* | 29 (26-33) | 26 (24-32) |  | 25 (21-30) | 23 (20-28) |  | 17 (10-19) | 16 (11-18) |  |
|  | p=0.05 | p=0.15 |  | p=0.90 | p=0.47 |  | p=0.27 | p=0.11 |  |
| Thought about ending life in past month? |  |  |  |  |  |  |  |  |  |
| No* | 28 (24-31) | 26 (22-30) |  | 25 (21-29) | 24 (19-28) |  | 15 (12-17) | 14 (11-16) |  |
| Yes* | 28 (26-32.5) | 26 (24-31) |  | 25 (22-27) | 24 (20-27) |  | 16.5 (14.5-19) | 16 (12-18) |  |
|  | p=0.24 | p=0.28 |  | p=0.81 | p=0.87 |  | p=0.03 | p=0.08 |  |
| Disclosed abuse to someone? |  |  |  |  |  |  |  |  |  |
| No* | 28.5 (25-32) |  |  | 25 (21-30) |  |  | 15 (12-18) |  |  |
| Yes* | 26 (23-29.5) |  |  | 25 (20.5-28) |  |  | 16 (12-17) |  |  |
|  | p=0.002 |  |  | p=0.59 |  |  | p=0.87 |  |  |
|  |  |  |  |  |  |  |  |  |  |
| Depressive symptoms (CES-D)** | 0.082 | 0.124 | 0.10*** | 0.006 | 0.145 | 0.04 | 0.049 | 0.075 | 0.13 |
|  | p=0.34 | p=0.01 |  | p=0.63 | p=0.004 |  | p=0.32 | p=0.41 |  |
| At risk of clinical depression (CES-D≥16) |  |  |  |  |  |  |  |  |  |
| No* | 28 (24-31) | 25 (22-30) |  | 25 (21-29) | 24 (19-27) |  | 15 (12-17) | 14 (11-16) |  |
| Yes* | 28 (24-31) | 26 (24-31) |  | 25 (21-30) | 25 (22-28) |  | 16 (12-19) | 15 (12-17) |  |
|  | p=0.53 | p=0.10 |  | p=0.49 | p=0.03 |  | p=0.09 | p=0.36 |  |
|  |  |  |  |  |  |  |  |  |  |
| Post-traumatic stress (HTQ)** | 0.143 | 0.078 | 0.07 | -0.021 | 0.068 | <0.001 | 0.093 | 0.164 | 0.11 |
|  | p=0.04 | p=0.05 |  | p=0.77 | p=0.12 |  | p=0.13 | p=0.13 |  |
| At risk of PTSD (Avg HTQ raw score ≥2.5) |  |  |  |  |  |  |  |  |  |
| No* | 27.5 (24-31) | 25 (23-30) |  | 25 (21-28.5) | 24 (19-27) |  | 15 (12-17) | 14 (11-16) |  |
| Yes* | 28 (26-32) | 27 (24-32) |  | 25 (19.5-30) | 27 (22-31) |  | 16 (12-18.5) | 16 (14-17) |  |
|  | p=0.10 | p=0.07 |  | p=0.76 | p=0.008 |  | p=0.42 | p=0.002 |  |

* Median and IQR, P-values obtained from t-test if distribution parametric, or Wilcoxon rank-sum test if distribution non-parametric

** Spearman’s rank correlation coefficient (ρ) and significance test of independence

*** P-value estimate presented is interaction term of regression analysis

## **Stata code for imputing missing values using mean of each candidate variable within respondent strata based on physical or sexual IPV survivorship**

* generate a set of variables with prefix 'm' who have missing values replaced by mean imputed variables (with sub strata of physexipv_ever

local implist womobey womobeyi moneyhus moneyhu1 mansay mansayi womperm wompermi norefsex norfsexi children childrni mangf mangfi punshwif unshwifi lobola lobolai lobolsex lobolsxi lovebeat lovebeti rapeblam rapeblmi wantrape wantrapi norape norapei hbrape hbrapei promisc promisci sexdo desbeat pregresp readysex condom strong embarr

foreach var of local implist {

ttest `var', by(physexipv_ever)

scalar mu0 = r(mu_1) // use ceil() to round the output

scalar mu1 = r(mu_2)

gen m`var' = `var'

tab m`var' physexipv_ever, mis

replace m`var'=ceil(mu0) if missing(`var')&physexipv_ever==0

replace m`var'=ceil(mu1) if missing(`var')&physexipv_ever==1

tab `var' physexipv_ever, mis

tab m`var' physexipv_ever, mis

}
